# The RXR Agonist MSU42011 Is Effective for the Treatment of Preclinical HER2+ Breast Cancer and Kras-Driven Lung Cancer

**DOI:** 10.3390/cancers13195004

**Published:** 2021-10-06

**Authors:** Ana S. Leal, Jessica A. Moerland, Di Zhang, Sarah Carapellucci, Beth Lockwood, Teresa Krieger-Burke, Bilal Aleiwi, Edmund Ellsworth, Karen T. Liby

**Affiliations:** 1 Department of Pharmacology and Toxicology, Michigan State University, East Lansing, MI 48824, USA; mendesle@msu.edu (A.S.L.); moerlan2@msu.edu (J.A.M.); zhangdi7@msu.edu (D.Z.); carapel1@msu.edu (S.C.); lockwo11@msu.edu (B.L.); kriege29@msu.edu (T.K.-B.); aleiwibi@msu.edu (B.A.); ellswo59@msu.edu (E.E.); 2 In Vivo Facility, Michigan State University, East Lansing, MI 48824, USA; 3 Medicinal Chemistry Facility, Michigan State University, East Lansing, MI 48824, USA

**Keywords:** RXR agonist, tumor microenvironment, lung cancer, breast cancer, immunotherapy

## Abstract

**Simple Summary:**

Breast and lung cancers are the most diagnosed cancers in the United States. Despite advances in treatment, over 176,000 deaths are expected in 2021, highlighting the need for new therapies. We evaluated MSU42011, a new RXR receptor activator, in murine models of breast and lung cancer, and this agonist reduced the tumor burden in both models. The tumor microenvironment is composed of numerous immune cells that can inhibit or promote tumor growth. MSU42011 modulated T cells within the tumor, reducing tumor-promoting immune cells and increasing tumor-killing cells. MSU42011 in combination with immunotherapy (anti-PDL1 and anti-PD1 antibodies) also decreased tumor burden when compared with individual treatments in the lung cancer model.

**Abstract:**

(1) Background: Notwithstanding numerous therapeutic advances, 176,000 deaths from breast and lung cancers will occur in the United States in 2021 alone. The tumor microenvironment and its modulation by drugs have gained increasing attention and relevance, especially with the introduction of immunotherapy as a standard of care in clinical practice. Retinoid X receptors (RXRs) are members of the nuclear receptor superfamily and upon ligand binding, function as transcription factors to modulate multiple cell functions. Bexarotene, the only FDA-approved RXR agonist, is still used to treat cutaneous T-cell lymphoma. (2) Methods: To test the immunomodulatory and anti-tumor effects of MSU42011, a new RXR agonist, we used two different immunocompetent murine models (MMTV-Neu mice, a HER2 positive model of breast cancer and the A/J mouse model, in which vinyl carbamate is used to initiate lung tumorigenesis) and an immunodeficient xenograft lung cancer model. (3) Results: Treatment of established tumors in immunocompetent models of HER2-positive breast cancer and Kras-driven lung cancer with MSU42011 significantly decreased the tumor burden and increased the ratio of CD8/CD4, CD25 T cells, which correlates with enhanced anti-tumor efficacy. Moreover, the combination of MSU42011 and immunotherapy (anti-PDL1 and anti-PD1 antibodies) significantly (*p* < 0.05) reduced tumor size vs. individual treatments. However, MSU42011 was ineffective in an athymic human A549 lung cancer xenograft model, supporting an immunomodulatory mechanism of action. (4) Conclusions: Collectively, these data suggest that the RXR agonist MSU42011 can be used to modulate the tumor microenvironment in breast and lung cancer.

## 1. Introduction

Breast cancer is the most common cancer diagnosis and is responsible for over 42,000 deaths in the United States per year [1]. Targeted therapies have reduced cancer deaths, but human epidermal growth factor receptor 2 (HER2) positive- and triple-negative breast cancer (TNBC) are often deadly, mostly due to acquired resistance. Worldwide, lung cancer remains the foremost cause of cancer deaths. Overall survival in lung cancer patients is less than 20%, despite the recent promise of targeted therapies and immunotherapy [1]. Over the last decade, immune checkpoint inhibitors, antibodies against PD1 and PDL1, have emerged as a standard of care to treat lung cancers without actionable targets. Nevertheless, patient responses to immunotherapy vary from 20% to 40%, highlighting the need to develop effective new drugs to enhance efficacy and suppress resistance [2].

Immunotherapy, and more recently small molecules used in combination with immunotherapy, were developed to target the immune compartment of the tumor microenvironment [3,4,5]. Immune checkpoints are required to maintain homeostasis and to prevent the inappropriate activation of CD8 cytotoxic T cells. Cancers disrupt the regulation of immune checkpoints to elude eradication by the immune system [6]. Retinoid X receptor (RXR) agonists can modulate the tumor microenvironment in murine models, leading to increased anti-tumor and decreased pro-tumor immune populations [7,8,9,10].

The RXR belongs to the nuclear receptor superfamily and acts as a transcription factor following ligand binding [11,12]. RXR heterodimerizes with others in the nuclear receptors superfamily, including the retinoic acid receptor (RAR), vitamin D receptor (VDR), peroxisome proliferator-activated receptor (PPAR), or liver X receptor (LXR). RXR homo- or heterodimers regulate the function of monocytes, linking cellular metabolism and immune function [8,13]. RXR agonists can stimulate phagocytosis, dampen responses to viruses, and increase cytokine secretion [8,14]. These immunomodulatory properties of RXR make it a pharmacologically attractive addition to current cancer therapies [15].

In 1999, the first synthetic RXR agonist, bexarotene (Targretin), was granted FDA approval to treat patients with cutaneous T-cell lymphoma [16]. Bexarotene has been tested in clinical trials for both lung and breast cancer but was not granted approval for these cancers [17,18]. Bexarotene binds to RAR, leading to significant adverse effects [17]. Other RXR agonists, including LG100268 and IRX194204 [19,20], have been synthesized to improve pharmacokinetic properties and diminish side effects. LG100268 and IRX194204 are active in murine models of lung [21] and breast cancer [7]. However, reported side effects from early clinical trials and FDA approval for bexarotene have hindered their clinical development [15]. We recently reported structure–activity relationship studies of new RXR agonists [22]. These small molecules were screened for suppression of nitric oxide, which correlates with activity against preclinical lung cancer, and limited changes in the expression of sterol regulatory element-binding protein (SREBP), which correlates with the elevation of triglycerides, a reported side effect of RXR agonists. From these in vitro studies, MSU42011 was chosen for additional efficacy studies in a murine lung cancer model [22,23].

Infiltration of immune cells into tumors is a hallmark of cancer and resistance to treatment, both in lung and breast cancers [24,25,26]. Because of the immune modulatory effects of RXR in cancer and other diseases [27], we tested the effects of MSU42011 on modulation of the tumor microenvironment in two different immunocompetent murine models of cancer. In HER2+ MMTV-Neu mice, the MMTV promoter directs the expression of inactivated neu (ErbB2/HER-2) to the mammary gland [28]. In the A/J mouse model of lung carcinogenesis, vinyl carbamate induces *Kras* mutations and the infiltration of immune cells observed in human lung cancer [21,29,30,31,32]. Our data show that MSU42011 has anti-tumor properties in immunocompetent mice, but not in an immunodeficient xenograft lung cancer model. MSU42011 also increased cytotoxic CD8 T cell activity in MMTV-Neu and A/J mice. Moreover, the combination of MSU42011 and anti-PD1 and anti-PDL1 antibodies was more effective in the A/J lung cancer model.

## 2. Materials and Methods

### 2.1. Drugs

MSU42011 was synthesized (>95% purity) as described [22]. Bexarotene (>99% purity) was acquired from LC Laboratories.

### 2.2. Animal Experiments

All in vivo studies were compliant with animal protocols (20180050) approved by the Institutional Animal Care and Use Committee at MSU in accordance with AAALAC-accredited Standards for the Management of Laboratory Animals.

MMTV-neu mice (Jackson Laboratory, Bar Harbor, ME, USA) were bred at MSU. Female MMTV-neu mice were fed standard rodent chow until palpable tumors were detected. When tumors reached a size of 32–64 mm^3^, mice were randomized and fed either control 5002 diet or MSU42011 in 5002 diet (100 mg/kg diet) [33]. Tumor diameters were measured twice per week.

Female A/J mice (Jackson Laboratories, Bar Harbor, ME, USA) were injected i.p. with 0.32 mg vinyl carbamate (Toronto Research Chemicals, Ontario, Canada) at both 7 and 8 weeks of age. One week before initiation, mice were started on AIN-93G diet (BioServ, Flemington, NJ, USA), which continued throughout the study. Mice (*n* = 15/group) were randomized and treated for 12 weeks with 100 mg RXR agonist/kg AIN-93G diet (~25 mg/kg body weight). Anti-PDL1, anti-PD1, and isotype control antibodies (Biolegend Go Invivo, San Diego, CA, USA) were injected i.p. (50 µg per mouse, twice a week for 12 weeks), beginning 10 weeks after initiation. Lungs were harvested and then inflated with PBS. The left lobe was fixed in neutral-buffered formalin (NBF), embedded in paraffin, step-sectioned (2 sections/lung, 600 microns apart), and stained with Hematoxylin and Eosin. The slides were evaluated as described [21,22]. One-half of the right lung was evaluated by flow cytometry and the other half was flash frozen for mRNA analysis.

Male athymic nude mice (Envigo, >5–6 weeks of age) were injected in the flank with human A549 lung cancer cells (5 × 10^6^ cells/mouse, cell authenticity confirmed by IDEXX BioAnalytics prior to injection). Calipers were used to measure tumor diameter and when 4 mm in diameter, mice were randomized (*n* = 7/group) and injected i.p. M-F with vehicle control (DMSO:Cremophor EL:saline 1:1:8) or RXR agonists (bexarotene or MSU42011 25 mg/kg) and/or carboplatin (15 mg/kg in saline once per week). Tumor size was measured with calipers twice per week and mice were weighed once per week. Tumor volume = length × width × (length + width/2)/2. After 4 weeks, tumors and tissues were harvested.

### 2.3. Flow Cytometry

In MMTV-Neu mice, a third of the tumor was minced and incubated with collagenase (300 U/mL, Sigma, St. Louis, MO, USA), dispase (1 U/mL, Worthington, Lakewood, NJ, USA), and DNAse (2 U/mL, Calbiochem, Burlington, MA, USA) for 30 min at 37 °C with stirring. A single-cell suspension was obtained by passing the cell solution through a 40 µm cell strainer (BD Falcon), and red blood cells were eliminated using a lysis buffer. Single cells were resuspended in PBS/0.5% BSA/0.1% azide and stained at 4 °C with the following antibodies for 30 min: CD45-VioGreen (30F11, Miltenyi, Bergisch Gladbach, Germany, 10:100), Gr-1-PE (RB6-8C5, Miltenyi, Bergisch Gladbach, Germany, 10:100), CD11b-FITC (M1/70.15.11.5, Miltenyi, Bergisch Gladbach, Germany, 10:100), CD19-APC (1D3/CD19, Biolegend, San Diego, CA, USA, 1:100), B220-PerCP-Cy5.5 (RA3-6B2, Biolegend, San Diego, CA, USA, 1:100), CD3-PE (145-2C11, Biolegend, San Diego, CA, USA 1:100), CD4-FITC (Gk1.5, Miltenyi, Bergisch Gladbach, Germany, 10:100), CD8-APC (53–6.7, Biolegend, San Diego, CA, USA, 1:100), CD25-PE.Cy7 (EBiosciences, San Diego, CA, USA, 1:100), and 5 μg/mL anti-mouse CD16/CD32 antibody (Biolegend, San Diego, CA, USA,) to reduce antibody binding to Fc receptors. Propidium iodide staining was used to exclude dead cells. Cells were analyzed using a BD FACS ARIA (BD) with three laser sources (488 nm, 633 nm, and 405 nm) and FlowJo x.10.0.7r2 software (Tree Star). Our gating strategy has been published [7].

Two lobes of the right lung were harvested from each A/J mouse (*n* = 6/group) for flow cytometry and incubated in digestion media and processed as described for the MMTV-Neu mice but with the following antibodies: CD45-Brilliant violet 510 (30F11), CD24-Brilliant violet 604 (M1/69), CD64-Brilliant violet 711 (X54-5/7.1), AI/AE-Brilliant violet 650 (M5/114.15.2), CD8-Alexafluor 700 (53–6.7), CD4-FITC (GK1.5), LyC6/LyG6-APC (rb6-8C5), CD11b-PE-Cy7 (M1/70), CD11c-PE-Cy5 (N418), CD206-Brilliant violet 421 (C068C2), CD69-Brilliant violet 785 (41.2F3), CD25-PE (3C7), and CD107a-Alexafluor 647 (1D4B). Zombie NIR staining was used to exclude dead cells. Cells were analyzed using a Cytek Aurora equipped with 4 lasers (405 nm, 488 nm, 561 nm, and 640 nm) and FlowJo x.10.0.7r2 software (Tree Star). Our gating strategy has been published [22].

### 2.4. Immunohistochemistry

A third of the tumor from MMTV-neu or the left lung from A/J mice were fixed in NBF for 48 h, embedded in paraffin, and sectioned. Endogenous peroxidase was quenched by hydrogen peroxide. Sections were immunostained with the following antibodies: CD206 (1:200, Abcam, Cambridge, United Kingdom), CD4 (1:40, GK1.5, Biolegend, San Diego, CA, USA), CD8 (1:40, 53–6.7, Biolegend, San Diego, CA, USA), FOXP3 (1:50, FJK-16s, EBiosciences, San Diego, CA, USA), PD-1 (1:100, EPR20665, Abcam, San Diego, CA, USA), p-ERK (1:100, 5A1E, Cell Signaling, Danvers, MA, USA), PCNA (1:200, sc-56, Santa Cruz Biotechnologies, Dallas, TX, USA), and F4/80 (1:50, Abcam, Cambridge, United Kingdom) and visualized with biotinylated anti-rabbit or anti-rat secondary antibodies (Cell Signaling or Vector Labs). Signal was detected using a DAB substrate (Cell Signaling) following the manufacturer’s recommendations. Sections were counterstained with hematoxylin (Vector Labs).

### 2.5. Ultrasound

Fur was removed over the thoracic cavity using the Nair chemical depilatory. Mice were anesthetized with isofluorane at 0, 4, and 12 weeks of treatment. A Vevo 2100 (Fujifilm VisualSonics, Toronto, ON, Canada) high-frequency ultrasound with a MS550D 40 MHz transducer was used to image the lung tumors [22].

### 2.6. Lipid Levels in Plasma

Plasma (*n* = 8 mice/group) harvested at necropsy was used to measure triglyceride and cholesterol level using commercial kits (Triglyceride Quantification Assay Kit from Abcam) and a Cholesterol Quantification Kit from Sigma-Aldrich.

### 2.7. Patient Survival Analysis

RXRα was used to analyze relapse-free survival and compare tumor vs. adjacent normal tissue. Aggregate data from KMPlot (http://www.kmplot.com, accessed on 6 January 2021) was auto-selected for the best cutoff (25th and 75th percentiles). Ethical approval was not required due to the retrospective nature of this study.

### 2.8. Statistical Analysis

Results were expressed as the mean ± standard deviation or the mean ± standard error as indicated in the respective figures/tables. All in vitro data were normally distributed and thus were analyzed using one-way ANOVA. The Tukey HSD multiple comparison method was used to evaluate changes between groups (VassarStats.com, accessed on 1 April 2021). Animal data were analyzed using SigmaStat 3.5 and one-way ANOVA. If data were normally distributed, the Holm–Sidak test for multiple comparisons was used. If data were not normally distributed, a Kruskal–Wallis one-way ANOVA on ranks and a Dunn test for multiple comparisons were used. Histological grades were analyzed using a McNemar’s Z test. For all studies, *p* < 0.05 was required for significance.

## 3. Results

### 3.1. RXR Expression Correlates with Increased Survival in Lung and Breast Cancers

**Figure 1:**
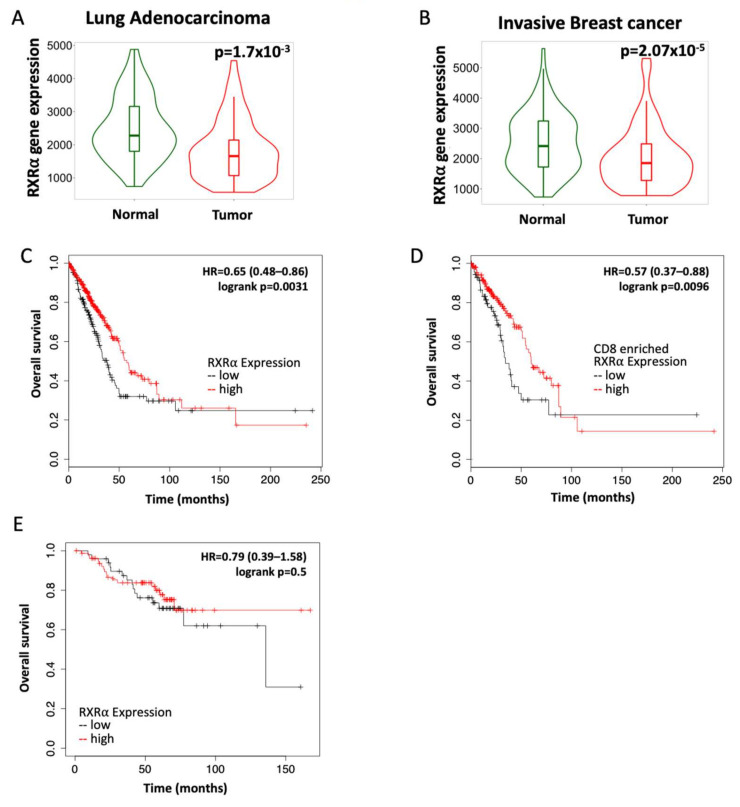
RXR expression correlates with overall survival in patients with lung adenocarcinoma and HER2-positive breast cancer. RXRα expression was evaluated by RNA sequencing in tumors and adjacent normal tissue from patients with (**A**) lung adenocarcinoma (*n* = 57) or (**B**) invasive breast cancer (*n* = 112). Prognostic value of high vs. low RXRα expression in patients with (**C**) lung adenocarcinoma (*n* = 504; 330 high and 174 low); (**D**) lung adenocarcinomas enriched for CD8 T cells (*n* = 273; 203 high and 72 low) and (**E**) HER2-positive breast cancer (*n* = 129; 80 high and 49 low). Data for all plots were accessed and analyzed using KMPLOT.

The RXR nuclear receptor is expressed in all tissues in the body, as RXR regulates mechanisms required for tissue maintenance such as glucose consumption, cell division, and immune regulation [12,27]. Because RXR is essential in normal physiology, we hypothesized that RXR expression will be downregulated in cancer. Using KMPLOT, a publicly available database, the expression of RXR in paired samples of tumors vs. normal adjacent tissue was analyzed [34,35]. In lung adenocarcinomas, the level of RXRα mRNA was significantly (*p* = 0.0017) lower in the cancer tissue compared to adjacent normal tissue (Figure 1A) in the 57 paired samples available. In breast cancers, RXRα mRNA expression was significantly (*p* = 0.0000207) lower in the tumor samples than in adjacent normal tissue in 112 paired samples from advanced breast cancer patients (Figure 1B) [34]. This trend was also observed in other cancer types and for the RXRβ isoform (Figure S1). 

Because tumor samples had lower RXRα expression, we interrogated whether the expression of RXR could predict survival in breast and lung cancer patients [35]. In lung adenocarcinomas, a high expression of RXRα significantly (*p* = 0.0031) correlated with the probability of increased survival. Overall survival was 58 months in patients with high RXRα expression compared to approximately 38 months in patients with low RXRα expression (Figure 1C). When samples from lung adenocarcinoma patients were selected for enrichment in CD8 cytotoxic T cells, the overall survival was 60 months (five years) in patients with the highest expression vs. 34 months in patients with the lowest expression (*p* = 0.0096; Figure 1D). In HER2-positive breast cancer, there was no significant difference in overall survival, although a trend toward extended long-term survival was found in the subset with high RXRα expression (Figure 1E). These observations suggest that targeting and activating RXR could be beneficial to patients with lung and breast cancer.

### 3.2. MSU42011 Reduced Tumor Growth in the MMTV-Neu Model of Breast Cancer and the A/J Mouse Model of Lung Cancer

We and others have shown that RXR agonists can prevent or treat HER2-positive and Kras-driven murine models of breast and lung cancers [7,21,22,23,36,37,38,39]. To evaluate the anti-tumor activity of the novel RXR agonist, MSU42011, the same models were used.

**Figure 2:**
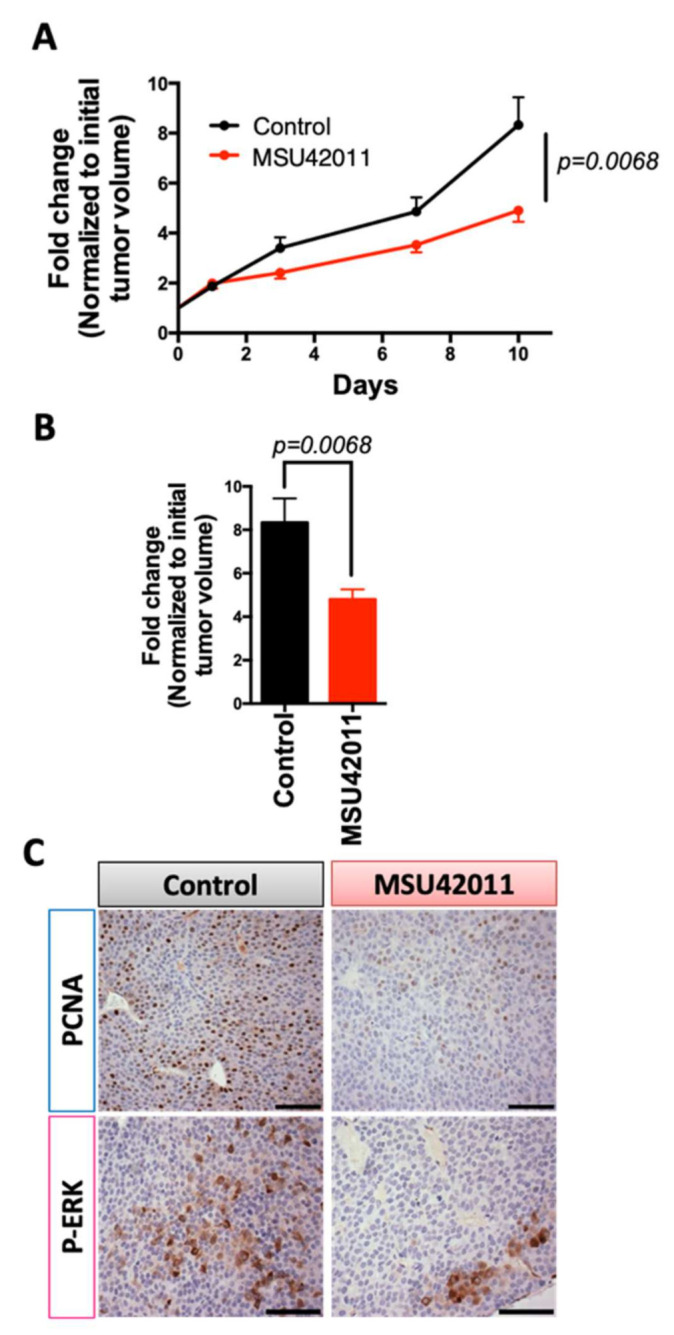
MSU42011 decreases tumor burden in MMTV-Neu mice, a model of HER2-positive breast. MMTV-Neu mice with tumor volumes at least 32 mm^3^ in volume were randomized and treated with either control diet or MSU42011 (100 mg/kg diet or ~25 mg/kg body weight) for 10 days (*n* = 20/group). (**A**) Fold change in tumor volume over a 10-day period or (**B**) fold change difference on the final day of treatment. (**C**) Cell proliferation in the mammary gland tumors was evaluated by immunostaining for proliferating cell nuclear antigen (PCNA) and p-ERK (scale bar 30 µm).

The MMTV-Neu model of HER2-positive breast cancer is driven by targeted expression by the MMTV promoter of the *Neu* (also known as *ErbB2*) oncogene to the mammary gland [28]. HER2/neu is a tyrosine kinase receptor shown to be overexpressed in >20% of breast cancers. Patients with HER2-positive breast cancer are treated with trastuzumab (Herceptin), pertuzumab, or new antibody–drug conjugates [40,41,42]. Trastuzumab is effective for treating tumors in MMTV-Neu mice, which validates the clinical relevance of the model [28]. MMTV-Neu mice with tumor volumes of 32 mm^3^ were treated with MSU42011 (100 mg/kg diet, or approximately 25 mg/kg of body weight) for 10 days [7]. MSU42011 significantly (*p* = 0.0068) decreased both tumor growth (Figure 2A) and final tumor volume when compared to the initial volume (Figure 2B and Figure S2A,B) vs. control mice. Fold changes were utilized because of variability in tumor growth rates between mice (individual tumor growth shown in Figure S2C) as there was no difference in volume at the time treatment was initiated (Figure S2A). No changes in plasma triglycerides or cholesterol levels were observed in the mice (Figure S2D). Consistent with the decreased tumor growth, PCNA, a biomarker of proliferation, and p-ERK staining were also reduced in tumors in mice treated with MSU42011 (Figure 2C and Figure S2E). HER2/neu triggers intracellular signaling, including the activation of the mitogen-activated protein kinases (MAPK) and consequently ERK phosphorylation, which is linked to proliferation and apoptosis [43,44].

**Figure 3:**
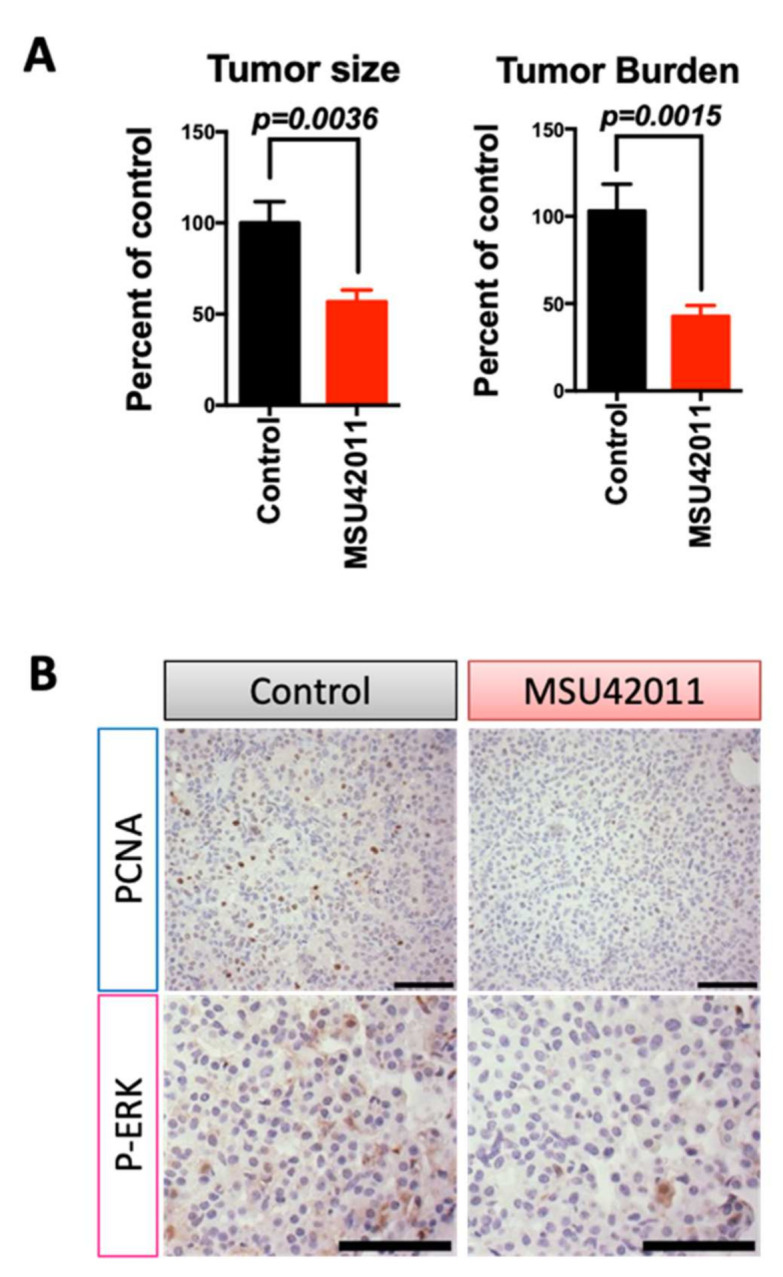
MSU42011 decreases tumor size and burden in AJ mice, a model of lung adenocarcinoma. Female A/J mice were injected with 2 doses of vinyl carbamate, 1 week apart, to initiate lung carcinogenesis. After 8 weeks, mice were fed either control AIN-93G diet or MSU42011 in the same diet (100 mg/kg diet). (**A**) Mice were euthanized 12 weeks after starting diet, and tumor size and burden were evaluated on lung sections (*n* = 14–15/group). (**B**) Cell proliferation in the lung tumors was evaluated by immunostaining for PCNA (proliferating cell nuclear antigen). (scale bar 30 µm).

To evaluate efficacy in a preclinical lung cancer model, A/J mice were challenged with the carcinogen vinyl carbamate, which causes *Kras* mutations and subsequent lung cancer [23,45]. At eight weeks after initiation, mice were treated with a control diet or MSU42011 diet (100 mg/kg diet). After 12 weeks of treatment, the lungs were harvested and the samples blinded and randomized prior to analysis. Mice in the MSU42011 treatment group showed a significantly reduced tumor size (*p* = 0.0036) and tumor burden (total tumor volume/number of lung sections; *p* = 0.0015) vs. the control group (Figure 3A, Table 1). The average tumor number and burden were 2.7 ± 0.4 tumors and 0.43 ± 0.06 mm^3^ in the MSU42011 group vs. 3.7 ± 0.4 tumors and 1.03 ± 0.25 mm^3^ in the control group, a 27–58% reduction. The percentage of tumors graded low or medium was significantly (*p* < 0.001) lower in the lungs of mice fed MSU42011 vs. control (48 vs. 34%, respectively). Bexarotene is inactive in this model [22]. We observed a decrease of PCNA and p-ERK, a protein downstream of Kras, in the lungs of mice treated with MSU42011 (Figure 3B and Figure S3A), similar to the reduction documented in the MMTV-Neu mice (Figure 2B). Triglycerides and cholesterol were not elevated by MSU42011 at the time of necropsy, and no changes in the total body weight were observed between groups at the time of the necropsy (Figure S3B).

### 3.3. MSU42011 Induced an Anti-Tumor Immune Response in the Tumor Microenvironment

We previously reported that the RXR agonist LG100268 favorably modulated the tumor microenvironment in the MMTV-Neu HER2-positive model [7]. Moreover, RXR plays important roles in several diseases where aberrant immune cell activation drives disease pathogenesis and pharmacological activation of RXR modulates immune cells [27]. To understand the effects of MSU42011 on the tumor microenvironment, immune cell populations in mammary tumors from MMTV-Neu mice and lungs from A/J mice were profiled by flow cytometry.

**Figure 4:**
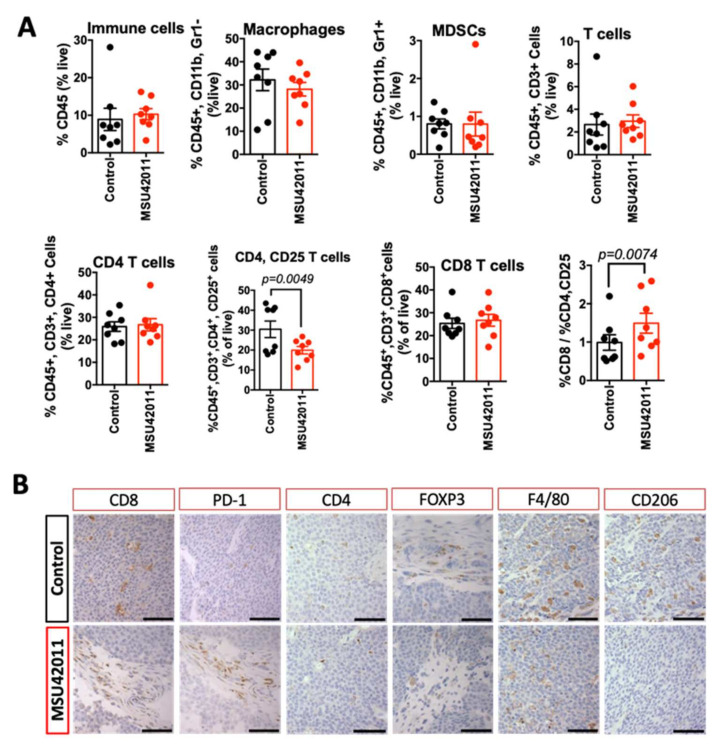
MSU42011 increases the percentage of activated CD8 cytotoxic T cells in the MMTV-Neu model of HER2-positive breast cancer. (**A**) Immune cell analysis by flow cytometry of whole tumor lysates (*n* = 8/group) or (**B**) immunohistochemistry for tumors from MMTV-Neu mice treated for 10 days with MSU42011 or control diet. (scale bar 30 µm).

Analysis of mammary tumors exhibited no significant changes in total immune cells, macrophages, myeloid-derived suppressor cells (MDSCs), and total T cells (including CD4 and CD8 T cells) present in the tumors (Figure 4A). However, activated CD4 T cells (the percentage of CD4, CD25 T cells) were significantly (*p* = 0.0049) lower in tumors of mice treated with MSU42011 compared to control mice (20 vs. 30% of CD4 T cells, respectively). CD4, CD25 T cells are associated with a poor prognosis in breast cancer and correlate with the expression of FOXP3, a tumor-promoting CD4 T cell, also termed regulatory T cells [46,47]. Consistent with the decrease in the percentage of the CD4, CD25 population in tumors from MMTV-Neu mice, we observed a trend toward decreased FOXP3 expression, shown by immunohistochemistry staining (Figure 4B). Because CD4, FOXP3^+^ T cells tend to aggregate in tumors, qPCR showed variability (Figure S4A) depending on the tumor section used so we confirmed the decrease in FOXP3 mRNA expression in T cells sorted from tumors (Figure S4B, *p* = 0.0072). To investigate if the decrease in FOXP3 expression was a direct effect of MSU42011 on CD4 T cells, isolated CD4 T cells from murine spleens were stimulated with TGFβ and IL-2 to induce FOXP3 and then treated with the drug for four days. MSU42011 (100 nM) decreased the levels of FOXP3 mRNA expression in CD4 T cells in vitro by 30% (Figure S4C).

The decrease in CD4, CD25 T cells complemented a higher ratio of the percentage of CD8:CD4, CD25 T cells (*p* = 0.0074), which correlates with increased anti-tumor activity by CD8 T cells [48]. The increase in the cytotoxic capability of CD8 T cells resulted in significantly (*p* = 0.0026) higher levels of interferon-gamma (IFNγ) mRNA in whole tumor lysates (Figure S4A). To determine if the increased IFNγ in whole tumors was derived from T cells (CD3) or myeloid cells (CD11B), a single cell suspension from tumors was sorted by magnetic separation beads and the resulting fraction was analyzed by qPCR. Both CD11B and CD3 cells isolated from tumors from mice treated with MSU42011 had increased IFNγ expression. The increase in IFNγ mRNA in the CD11B population prompted us to interrogate if the macrophages present had changed to a less tumor-promoting phenotype since analysis by flow cytometry showed no change in the percentage of the CD11B population infiltrating into the tumors (Figure 4A). Immunohistochemistry of tumors showed a marked decrease in the expression of CD206 (Figure 4B), a marker of tumor-promoting macrophages [49].

**Figure 5:**
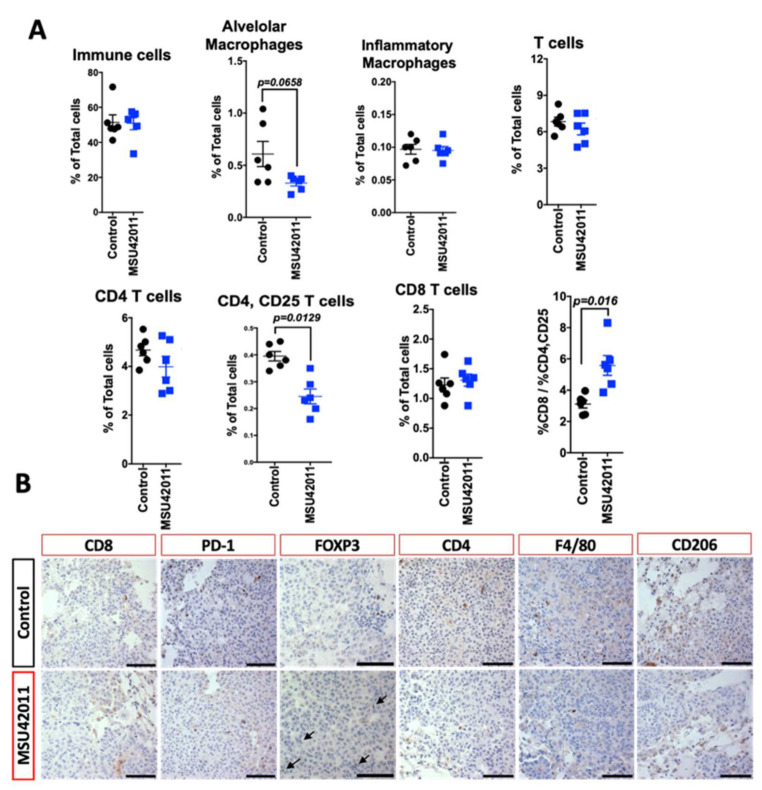
MSU42011 increases the percentage of activated CD8 cytotoxic T cells and decreases macrophages in the AJ model of lung carcinogenesis. (**A**) Flow cytometry analysis of whole lung lysates from A/J mice injected with vinyl carbamate to induced lung cancer (*n* = 6/group). (**B**) Immunohistochemistry of tumor sections to confirm the flow cytometry data. (scale bar 30 µm).

To determine if similar effects were observed in the lung cancer model, immunoprofiling was conducted on the lungs of A/J mice on the control or treatment diet. Flow cytometry revealed a trend toward a decrease in alveolar macrophages (*p* = 0.0658), but we observed no changes in other immune cell populations in the lung, including monocytes or T cells (Figure 5A). As previously reported [22] and as observed in the MMTV-Neu tumors (Figure 4B), MSU42011 treatment decreased CD206 macrophage expression (Figure 5B). Analysis of T cell activation revealed that the percentage of CD4, CD25 T cells was decreased in the lungs of mice treated with MSU42011 (*p* = 0.0129 vs. control, 0.4% vs. 0.22%). This decrease was accompanied by a trend toward decreased FOXP3 staining within the tumors (Figure 5B), although no significant change (*p* = 0.3) in IFNγ mRNA levels in lung lysates was observed (Figure S5). The percentage of CD8 T cells infiltrating into the lungs of A/J did not change when treated with MSU42011. However, because of the decrease in activated CD4, CD25 T cells, the ratio of CD8: CD4, CD25 was increased in mice treated with MSU42011 (*p* = 0.016, 3 vs. 6). The increase in the ratio of CD8:CD4, CD25 is characteristic of increased anti-tumor CD8 T cells [50].

### 3.4. MSU42011 Was Ineffective for Suppressing Tumor Growth in a Human A549 Lung Cancer Xenograft Model

**Figure 6:**
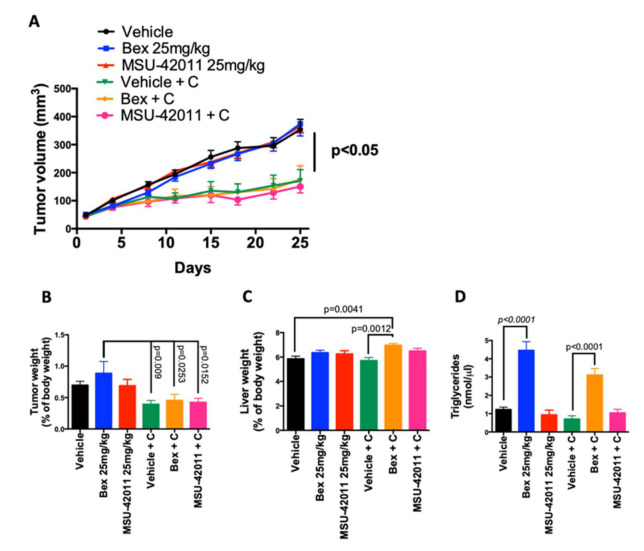
RXR agonists do not inhibit tumor growth in an A549 xenograft cancer model. Male nude mice were injected in the flank with human A549 lung cancer cells. When tumors reached 4 mm in diameter, mice were treated for 4 weeks with either vehicle control or RXR agonists [25 mg/kg bexarotene (bex) or MSU42011] 5 days per week, alone or in combination with carboplatin (C, 15 mg/kg) once per week. Tumors were measured with calipers two times per week. *n* = 7 mice per group (**A**) Evolution of A459 xenograft tumor growth over time. (**B**) Tumor and (**C**) liver weights (*n* = 7/group) and (**D**) triglycerides levels in plasma (*n* = 5) at the time of necropsy.

Xenograft models are widely used to test potential new cytotoxic therapeutics [51,52]. However, RXR agonists lack apoptotic and/or anti-proliferative activity against cancer cells in vitro [7,22]. To test the efficacy of MSU42011 and bexarotene against human lung cancer cells in vivo, A549 lung cancer cells were injected subcutaneously into the flank of nude mice. Once tumors grew to a size of 4 mm in diameter, mice were treated with either bexarotene or MSU42011 (25 mg/kg) by i.p. injection five times per week for 20 days. Neither bexarotene nor MSU42011 slowed tumor growth (Figure 6A and Figure S6A), and at the time of necropsy, no differences in tumor weights were found between individual treatment groups (Figure 6B).

Lung cancer patients with *KRAS* mutations are commonly treated with chemotherapy. To test if the A549 lung tumors would respond to chemotherapy and if the combination with bexarotene or MSU42011 would enhance efficacy, mice were treated orally five times a week and carboplatin was added as an intraperitoneal injection once a week (15 mg/kg). All groups treated with carboplatin had a significant (*p* < 0.05) reduction in tumor growth over time (Figure 6A and Figure S6A) and reduced tumor weight at the time of necropsy (Figure 6B) when compared with the groups not receiving carboplatin. However, the combination of carboplatin and MSU42011 or bexarotene was not more effective than the chemotherapy alone. We observed no changes in total body weight across groups at the time of necropsy (Figure S6B).

We and others have previously reported that RXR agonists can induce liver hypertrophy [21,22], leading to increased liver weight and hyperlipidemia due to engagement of the RXR-LXR heterodimer. Our medicinal chemistry strategy minimized these effects with MSU42011 [22]. In this cohort, only mice treated with bexarotene plus carboplatin showed a significant (*p* = 0.0012) increase in liver weight, when compared with mice receiving carboplatin alone or the vehicle control (Figure 6C). Moreover, bexarotene alone or in combination with carboplatin significantly (*p* < 0.001) increased the levels of triglycerides in plasma, compared with vehicle or carboplatin alone (Figure 6D). The same pattern was also observed for cholesterol levels (Figure S6C). In contrast, MSU42011 did not increase either triglycerides or cholesterol in this model. The lack of effect on tumor growth in the A549 xenograft model with both bexarotene and MSU42011 suggests that RXR agonists require a functional immune system for anti-tumor activity, which is lacking in xenograft models.

### 3.5. MSU42011 in Combination with Anti-PD1 or Anti-PDL1 Antibodies Was Effective for Treating Established Lung Tumors in the A/J Mouse Model

Because we observed an increase in functional anti-tumor CD8 T cells (increased ratio of CD8: CD4, CD25) we interrogated if the combination of MSU42011 with anti-PD1 or anti-PDL1 antibodies would be beneficial in the A/J lung cancer model. Tumors were initiated with vinyl carbamate as described above and a diet containing MSU42011 (100 mg/kg) was started eight weeks post-carcinogen. Anti-PD1 and anti-PDL1 antibodies were given twice a week by intraperitoneal injection at 50 μg/mouse, starting two weeks after the mice were started on a treatment diet, for a total of 22 doses. Isotype control antibodies plus a control diet or MSU42011 diet were used as control groups. This treatment regimen was well-tolerated, with no signs of weight loss or increased triglycerides or cholesterol in plasma (Figure S7).

**Figure 7:**
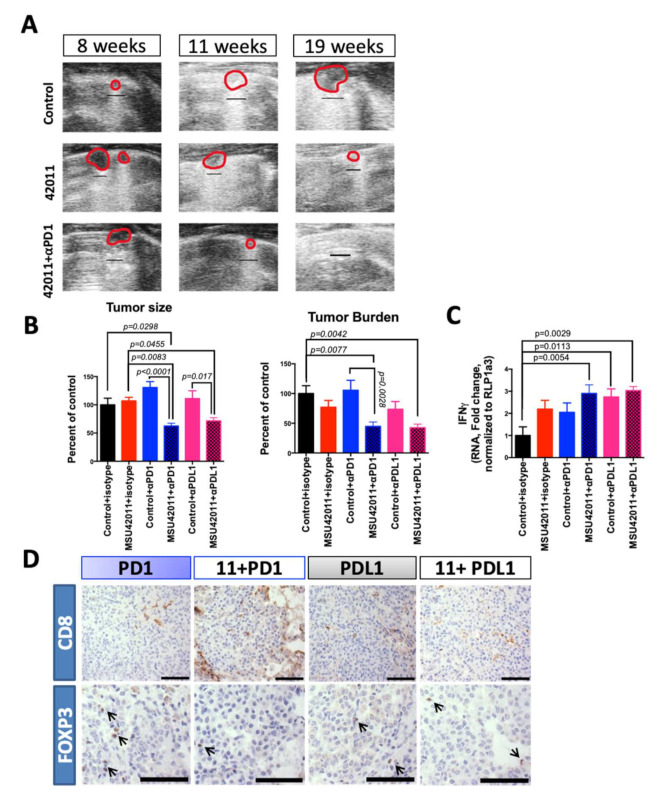
Combination of MSU42011 and anti-PD1 antibodies decreases tumor burden and increases anti-tumor T cells in the AJ model of lung cancer. (**A**) Ultrasound imaging of lung tumor growth over time in A/J mice challenged with vinyl carbamate to induce lung tumorigenesis in mice receiving only control diet, diet with MSU42011 (100 mg/Kg) or MSU42011 and anti-PD1 antibody. (**B**) Graphical representation of Table 2 for tumor burden and size of A/J mice treated with MSU42011 (100 mg/Kg-diet) plus anti-PD1 or anti-PDL1 antibodies. Mice were started on diet 8 weeks after vinyl carbamate injection, and anti-PD1, anti-PDL1 or isotype control antibodies were started 2 weeks after diet was started and given twice weekly (50 μg/mouse) for a total of 22 administrations. (**C**) RT-PCR analysis of whole lungs of A/J after treatment, for levels of IFNγ (*n* = 5). (**D**) Immunohistochemistry for CD8 and FOXP3 of lung tumors in A/J mice treated with anti-PD1 or anti-PDL1 antibodies in combination with MSU42011 (11). (scale bar 30 µm).

We previously reported that the evolution of lung tumors on the surface of the lungs can be visualized in living mice using a high-frequency ultrasound [22]. Eight weeks after the injection of vinyl carbamate, A/J mice were imaged (baseline) prior to starting treatment. Tumors 0.4 mm in diameter could be identified as they exhibited reduced echogenicity with hyperechogenic borders. Mice were imaged after 4 and 12 weeks of treatment, which equates to 11 and 19 weeks after initiation (Figure 7A). While tumors in the control group continued to increase in size over time, tumor size decreased in the lungs of mice treated with MSU42011. In mice treated with the combination of MSU42011 and anti-PD1 antibody, tumors also decreased in size, with some tumors below the level of detection following eight weeks of treatment. Additionally, the lung parenchyma surrounding tumors appeared less aerated in the control animals, with the disappearance of horizontal A-lines and the appearance of B-lines, usually related to the presence of interstitial edema [53]. In contrast, the lung architecture in the treatment groups appeared more normal, with A-lines remaining or reappearing upon treatment (Figure 7A).

The combination of MSU42011 plus anti-PD1 or anti-PDL1 antibodies significantly (*p* = 0.0493 and *p* = 0.0017, respectively) reduced the number of tumors per slide at necropsy when compared with the control plus isotype (71.7% and 59.5% of the control group respectively). The combination of MSU42011 plus anti-PD1 (130.7% vs. 62.5%, % of control, *p* < 0.0001) or anti-PDL1 (111.1% vs. 71.5%, % of control, *p* = 0.017) was more effective for reducing tumor size than antibodies alone (Table 2, Figure 7B) or the isotype control group. Average tumor burden was also reduced by more than 50% with the combination of MSU42011 plus anti-PD1 vs. control (0.46 ± 0.07 mm^3^ vs. 1.02 ± 0.13 mm^3^, *p* = 0.0077) or anti-PDL1 (0.43 ± 0.06 mm^3^, *p* = 0.0042) vs. control. Histologically, high grade lung tumors decreased from 68% in the control group to 46–48% in the combination groups (*p* = 0.077 and 0.043, respectively, Table 2). Similarly, low and medium grade tumors were significantly (*p* < 0.05) higher in the MSU42011 plus anti-PD1 or anti-PDL1 (52–54%) groups vs. the control group (32%).

Flow cytometry analysis of selected groups showed expected changes in immune cell profiles (Figure S8). As observed in the control vs. MSU42011 groups (Figure 5A), a decrease in CD4, CD25 T cells was detected in mice receiving anti-PDL1 antibodies and the combination of anti-PDL1 antibodies and MSU42011 (*p* = 0.0029 and *p* = 0.0403, versus control, respectively) (Figure S8). The reduction in CD4, CD25 T cells led to a significant increase in the ratio of CD8:CD4, CD25 T cells in the MSU42011 group and the combination group with anti-PDL1 when compared to the controls (3.1 vs. 5.6%, *p* = 0.016; 3.1 vs. 4.6%, *p* = 0.038) (Figure S8). The increase in the ratio of CD8:CD4, CD25 T cells predicts more active/anti-tumor cytotoxic CD8 T cells, and qPCR of whole lung lysates showed an increase in the expression of INFγ in all groups. However, only the MSU42011 plus anti-PD1, anti-PDL1, and MSU42011 plus anti-PDL1 groups showed significantly increased values (*p* = 0.0054, 0.0113, 0.0029, respectively) (Figure 7C). To further interrogate the spatial location of the CD8 T cells in the tumors, immunohistochemistry was performed. All groups receiving anti-PD1 or anti-PDL1 antibodies had CD8 T cells within the tumor, but only the combination with MSU42011 showed a reduction in the FOXP3 population of T cells within the tumors (Figure 7D). In summary, the combination of MSU42011 plus anti-PD1 or anti-PDL1 antibodies was efficacious in reducing tumor number, tumor size, and burden in the A/J mouse model of Kras-driven of lung cancer. Additionally, these combinations led to an increase in cytotoxic CD8 T cells with an increased capability to produce IFNγ.

## 4. Discussion

The only FDA-approved RXR agonist, bexarotene, has been tested in clinical trials for breast and lung cancers, unfortunately without conclusive clinical benefits [17], but the mutational burden of responders and the immunologic effects of this drug were never explored [39]. Our newest results support the hypothesis that the anti-tumor action of MSU42011, an RXR agonist, is dependent on a fully functional immune system, present in MMTV-Neu and A/J mice (Figure 2 and Figure 3), but not in athymic nude mice (Figure 6). Moreover, the reduction of lung cancer burden and size reported here is reproducible and similar in range to the reduction observed in an initial study with MSU42011 in the A/J mice [22]. RXR agonists were developed/repurposed with the expectation that they would reduce cancer cell proliferation and induce apoptosis and/or differentiation [37,54]. However, the immune regulatory effects of RXR agonists are important in diseases with a strong inflammatory component, such as viral infections, neurodegenerative diseases, and cancer [8,9,55,56].

Evidence of the relevance of the composition and activation status of the immune cells in the tumor microenvironment and their correlation to the likelihood of response to immunotherapy is a growing field [57,58,59]. Anti-PD1 and anti-PDL1 are now approved to treat lung cancer when targeted therapy is not available, such as in patients with *KRAS* mutations [60] that are not KRAS-G12C mutations, for which sotorasib is now approved. However, many patients do not qualify to receive immunotherapy because of the lack of expression of PD1 or PDL1, do not respond to therapy, or develop resistance [57,61]. The use of small molecules to modulate the tumor microenvironment, increase trafficking of CD8 cytotoxic T cells into the tumor, or alter cytokine production is one of the many strategies that has been developed to increase responses to immunotherapy [62]. MSU42011, in both the MMTV-Neu model of HER2-positive breast cancer and the A/J lung cancer model, increased anti-tumor CD8 T cells (Figure 4 and Figure 5), as seen by increased INFγ levels (MMTV-Neu, *p* = 0.0026), increased ratios of CD8:CD4, CD25 T cells, and decreased immunosuppressive FOXP3+ T cells (Figure 4 and Figure 5). This increase in anti-tumor CD8 T cell to CD4, CD25 T cell ratio led to a significant reduction in tumor size when MSU42011 was combined with both anti-PD1 or anti-PDL1 antibodies in the A/J lung cancer model when compared with MSU42011 or anti-PD(L)1 antibodies alone (Figure 7 and Table 2). The clinical use of anti-PD1 and anti-PDL1 antibodies is well known in lung cancer [61]. However, in breast cancer, anti-PDL1 antibodies were only recently approved to treat patients with triple-negative breast cancer [63]. To further evaluate the efficacy when combining MSU42011 with anti-PDL1 antibodies, we are currently testing this protocol in a murine model of triple-negative breast cancer. Targeting RXR can potentially increase the anti-tumor potential of anti-PD1 and/or anti-PDL1 antibodies without increasing toxicities (Figure S7).

The changes in T cell infiltration in both models only reflect small changes in the expression of FOXP3, both at the level of mRNA and protein expression (Figure 4B and Figure 5B and Figure S5). FOXP3 expression in cancer is usually associated with tumor-promoting CD4 T cells and the suppression of anti-tumor immune cells, such as effector CD8 T cells [64]. The expression of FOXP3 is a marker of terminal differentiation. However, experimental evidence has shown that immune suppression of FOXP3 expressing Treg cells can be decreased without the loss of FOXP3 expression [65]. Additionally, in a murine model of pancreatic cancer, depletion of FOXP3 expressing cells led to rapid disease progression when compared to mice with FOXP3 expressing cells [66]. These data suggest that MSU42011 can regulate immune suppressive features in FOXP3 T cells without significantly reducing FOXP3 expression. The full effects of RXR regulation in T cell physiology are likely context-dependent and vary in cancer versus autoimmune diseases [27,67]. Moreover, RXR function in T cells will vary in different cancer types, due to the cytokine and chemokine milieu.

The increased response to immunotherapy (anti-PD1 and anti-PDL1 antibodies) in this study is associated with the increased functionality of anti-tumor CD8 cells and a trend towards decreased tumor-suppressive FOXP3 T cells with the tumor microenvironment (Figure 7) [68,69]. CD3 and CD11B cells magnetically sorted from MMTV-Neu tumors showed increased expression of IFNγ (Figure S4B). Since CD11B can include macrophages and dendritic cells and both can produce IFNγ, it is possible that a feedback mechanism between myeloid cells and T cells is present when tumors are treated with MSU42011, leading to increased production of IFNγ from more than one cell type. Additionally, RXR activation, with both physiological or synthetic agonists, can increase the activation and recruitment of dendritic cells [70,71,72]. Dendritic cells are a key antigen-presenting cell, whose activation has been correlated with the recruitment and activation of anti-tumor CD8 cells [73,74] and more recently implicated in responses to immunotherapy [75,76]. Further studies are necessary to determine if RXR agonists increase the recruitment of CD8 T cells through the activation of dendritic cells and how this influences responses to immunotherapy.

*KRAS* mutations induce the formation of a highly immunosuppressive tumor microenvironment [2,77,78]. The KRAS pathway is activated in both models, either by an activating mutation in the A/J lung cancer model [29,30] or by activation of upstream signaling pathways in the MMTV-Neu model of HER2-positive breast cancer [28,43,44]. As a result, ERK phosphorylation is high in both models but MSU42011 reduced the expression of p-ERK in both murine models (Figure 2 and Figure 3). Targeting KRAS or downstream effectors, such as p-ERK, has been a goal for many years in the cancer research field with recent progress made with MAPK inhibitors and more recently with KRAS G12C inhibitors [79,80]. However, these inhibitors are toxic, ineffective against other *Kras* mutations (such as the Q61L mutation found in the A/J lung cancer model or the predominant G12D mutation found in pancreatic cancer), or have not been explored in the context of the tumor microenvironment [78]. The data reported here are encouraging since the RXR agonist MSU42011 not only decreased the expression of p-ERK and tumor-promoting immune populations but also simultaneously increased tumor-suppressive immune populations (Figure 4 and Figure 5).

## 5. Conclusions

In summary, we described the efficacy of MSU42011 in preclinical models of HER2-positive breast cancer and A/J lung cancer with an activating *Kras* mutation, its ability to increase anti-tumor immune cell populations in both animal models, and the ability of this novel RXR agonist to further decrease tumor burden when combined with anti-PD1 and anti-PDL1 antibodies. However, the complex interactions between cancer cells and immune cells within the tumor microenvironment and the modification of these interactions by RXR agonists require further inquiry. A greater understanding of the biology of RXR in the tumor microenvironment can increase pharmacological applications for these small molecules as immunomodulators and provide opportunities to increase the efficacy of immunotherapy [27,81,82].

## Figures and Tables

**Table 1:** The RXR agonist MSU42011 decreases tumor size and burden in a carcinogen-induced A/J model of lung cancer.

Groups Analyzed	Control	MSU42011 100 MG/KG DIET
**# SLIDES/GROUP**	30	28
**# TUMORS/GROUP**	110	75
**TOTAL # TUMORS/SLIDE** **(% OF CONTROL)**	3.67 ± 0.4 (100%)	2.68 ± 0.36 (73.1%)
**TOTAL # L AND M GRADE** **(% TOTAL)**	37 (34%)	36 (48%) (*p* < 0.001)
**TOTAL # H GRADE (% TOTAL)**	73 (66%)	39 (52%)
**TOTAL TUMOR VOLUME, MM^3^**	30.90	11.96
**AVE TUMOR SIZE (MM^3^)/TUMOR** **(% CONTROL)**	0.28 ± 0.033 (100%)	0.16 ± 0.02 (56.8%) (*p* = 0.0036)
**AVE TUMOR BURDEN (MM^3^)** **(% CONTROL)**	1.03 ± 0.15 (100%)	0.43 ± 0.062 (41.5%) (*p* = 0.0015)

**Table 2:** The combination of the RXR agonist MSU42011 and anti-PD1 or anti-PDL1 antibodies decreases tumor size and burden in carcinogen-induced model of lung cancer.

Groups Analyzed	CONTROL + ISO	MSU42011 + ISO	ANTI PD-1 AB	ANTI- PDL1 AB	MSU42011 + ANTI-PD1	MSU42011 + ANTI-PDL1
**# OF SLIDES/GROUP**	28	30	30	30	28	30
**# OF TUMORS/GROUP**	127	98	110	90	91	81
**TOTAL # TUMORS/SLIDE** **(% OF CONTROL)**	4.54 ± 0.46 (100%)	3.27 ± 0.4 (72%) ^a^	3.67 ± 0.33 (80.8%)	3.00 ± 0.27 (66.1%) ^b^	3.25 ± 0.35 (71.7%) ^c^	2.70 ± 0.25 (59.5%) ^d^
**TOTAL # L AND M GRADE (% TOTAL)**	40 (32%)	35 (36%)	42 (38%)	35 (39%)	49 (54%) ^e, f^	42 (52%) ^g^
**TOTAL # H GRADE** **(% TOTAL)**	87 (68%)	63 (64%)	68 (62%)	55 (61%)	42 (46%) ^h^	39 (48%) ^i^
**TOTAL TUMOR VOLUME, MM^3^**	28.54	23.536	32.304	22.48	12.78	13.01
**AVE TUMOR SIZE (MM^3^)/TUMOR** **(% CONTROL)**	0.22 ± 0.02 (100%)	0.24 ± 0.023 (106.9%)	0.29 ± 0.029 (130.7%)	0.25 ± 0.039 (111.1%)	0.14 ±0.014 (62.5%) ^j, k, l^	0.16 ± 0.016 (71.5%) ^m, n^
**AVE TUMOR BURDEN (MM^3^)**	1.02 ± 0.13 (100)	0.78 ± 0.11 (77%)	1.08 ± 0.17 (105.6%)	0.75 ± 0.13 (73.5%)	0.46 ± 0.073 (44.8%) ^o, p^	0.43 ± 0.061 (42.5%) ^q^

Female A/J mice were injected with vinyl carbamate and after 8 weeks, fed control diet or MSU-42011 in diet for 12 weeks +/− antibodies (Ab = antibody; iso = isotype control), as described in Figure 7. L = low, M = medium, H = high grade. ^a^: *p* = 0.0487 ( vs. C+iso), ^b^: *p* = 0.011 (vs. C+iso), ^c^: *p* = 0.0493 (vs. C+iso), ^d^: *p* = 0.0017 (vs. C+iso), ^e^: *p* = 0.046 (vs. C+anti-PD-1), ^f^: *p* = 0.028 (vs. MSU42011+iso), ^g^: *p* = 0.061 (vs. MSU42011+iso), ^h^: *p* = 0.077 (vs. MSU42011+iso), ^i^: *p* = 0.043 (vs. MSU42011+iso), ^j^: *p* = 0.0298 (vs. C+iso), ^k^: *p* = 0.0083 (vs. MSU-42011+iso), ^l^: *p* < 0.0001 (vs. anti-PD1), ^m^: *p* = 0.0455 (vs. MSU-42011+iso), ^n^: *p* = 0.017 (vs. anti-PDL1), ^o^: *p* = 0.0077 (vs. control + iso), ^p^: *p* = 0.0028 (vs. anti-PD1), ^q^: *p* = 0.0042 (vs. control + iso). Values represent mean ± SE. *p* values were calculated by ordinary one-way ANOVA and the posthoc Dunnetts multiple comparations test.

## Data Availability

For the meta-analysis cohort, we used aggregate data from KMPlot (http://www.kmplot.com, accessed on 6 January 2021) using auto-selection for best cutoff between the 25th and 75th percentiles.

## Supplementary Materials

The following are available online at https://www.mdpi.com/article/10.3390/cancers13195004/s1, Figure S1: Expression of RXRα (A) and RXRβ (B) in tumors vs. normal tissue, Figure S2: Tumor volume in MMTV-Neu mice treated with MSU42011, Figure S3: (A) Quantification of PCNA (*n* = 3) and p-ERK (*n* = 4) intensity staining by optical density (OD), Figure S4: Treatment with MSU42011 reduced FOXP3 and increased IFNγ, Figure S5: Evaluation of FOXP3 and IFNγ mRNA by MSU42011 treatment, Figure S6: Nude mice were injected s.c. with human A549 lung cancer cells, Figure S7: Final total body weight, levels of cholesterol and triglycerides in A/J mice treated with control, MSU42011, anti-PD1 or anti-PDL1 antibodies, or the combinations as described in Figure 7, Figure S8: Flow cytometry analysis of selected groups of A/J mice treated as described in Figure 7.
