# Supplementary material for: The RXR Agonist MSU42011 Is Effective for the Treatment of Preclinical HER2+ Breast Cancer and Kras-Driven Lung Cancer
Source: Cancers (Basel). 2021 Oct 6;13(19):5004. doi: 10.3390/cancers13195004 (PMC8508021; doi:10.3390/cancers13195004)
Supplement: Supplementary file 1 [file cancers-13-05004-s001.zip › cancers-1339130-supplementary.pdf]

# Supplementary Materials: The RXR Agonist MSU42011 Is Effective for the Treatment of Preclinical HER2+ Breast Cancer and Kras-Driven Lung Cancer

**Ana S. Leal, Jessica A. Moerland, Di Zhang, Sarah Carapellucci, Beth Lockwood, Teresa Krieger-Burke, Bilal Aleiwi, Edmund Ellsworth, Karen T. Liby**

**Supplementary Text**

## Supplemental methods

### CD4-FOXP3 T cell treatment

From a single cell suspension of splenocytes from a wild type mouse, $10^7$ cells were incubated in 100 μL of Mojo Buffer (Biolegend) with 10 μL of pre-diluted Biotin-antibody cocktail mix for 15 minutes, followed by 10 μL of pre-diluted streptavidin nanobeads for an additional 15 minutes on ice (Biolegend MojoSortTM CD4 T cell isolation kit). The mixture was then placed on the column in the magnetic separator and washed with 3 mL of Mojo Buffer. Isolated CD4 T cells were plated in a 24 well plate at 106 ml/well in RPMI media supplemented with 10% FBS, anti-mouse CD28 (clone 37.51, 3 μg/mL, Biolegend), recombinant IL-2 (5 ng/mL, Biolegend), and recombinant human TGFβ (5 ng/mL, R&D). Plates were coated with anti-mouse CD3ε (clone 145-2C11, 3μg/mL, Biolegend) over night at 4oC. After 24 hours, LG268 or bexarotene were added at 100 nM and CD4 T cells were cultured for an additional 4 days. On day 5 cells were collected and RNA was extracted using triZOL(Thermo Fisher Scientific) following the manufacturer's instructions. Two micrograms of RNA were reverse transcribed, and 1 μl of complementary DNA from this reaction was added to 12.5 μl of Bio-Rad iQ SYBRGREEN Supermix (Hercules, CA), 1 μl of validated RT2 quantitative PCR (qPCR) FOXP3 (F: 5′-CTC GTC TGA AGG CAG AGT CA -3′; R: 5′-TGG CAG AGA GGT ATT GAG GG -3′) or RLP13A (F: 5′-GTT GCC TTC ACA GCG TA-3′; R: 5′-AGA TGG CGG AGG TGC AG-3) primers and DNAse-free water for real-time qPCR. All expression data were normalized using RLP13A as the housekeeping control.

### CD11b and CD3 isolation

Tumors from MMTV-neu female mice were minced separately and incubated in digestion media consisting of collagenase (300 U/ml, Sigma), dispase (1 U/ml, Worthington), and DNAse (2 U/ml, Calbiochem) for 30 minutes at 37°C with stirring. Cells were then passed through a 40 μm cell strainer (BD Falcon). From a single cell suspension 107 cells were incubated in 100 μL of Mojo Buffer (Biolegend) with 10 μL of pre-diluted Biotin-antibody cocktail mix for 15 minutes, followed by 10 μL of pre-diluted streptavidin nanobeads for an additional 15 minutes on ice (CD11b cell isolation kit, Miltenyi). The mixture was then placed on the column in the magnetic separator and washed with 3 mL of Mojo Buffer. CD11b positive cells were then eluted from the column. Cells were lysed in RIPA buffer (1 M Tris-Cl, pH 7.4, 0.5 M EDTA, 5 M NaCl, 1% triton-X, 25 mM deoxycholic acid, 0.1% SDS) containing protease inhibitors (PMSF, aprotinin and leupeptin) for whole cell lysates.

CD3 T cells were isolated from the tumors using the same approach as described above for CD4.

### RT-qPCR analysis

Total RNA was isolated using the RNeasy Mini Kit (Qiagen, Valencia, CA). RNA concentrations were determined by NanoDrop, and 2 μg RNA was used to synthesize cDNA using SuperScript III reverse transcriptase (Invitrogen, Carlsbad, CA). Primers were ordered from IDT. iQ SYBR Green Supermix (Bio-Rad, Berkeley, CA) and the QuantStudio 7 Flex Real-Time PCR system were used to detect gene expression. The delta-delta Ct method was used to assess relative gene expression. Values were normalized to the reference gene GAPDH and expressed as fold change compared to saline control samples.

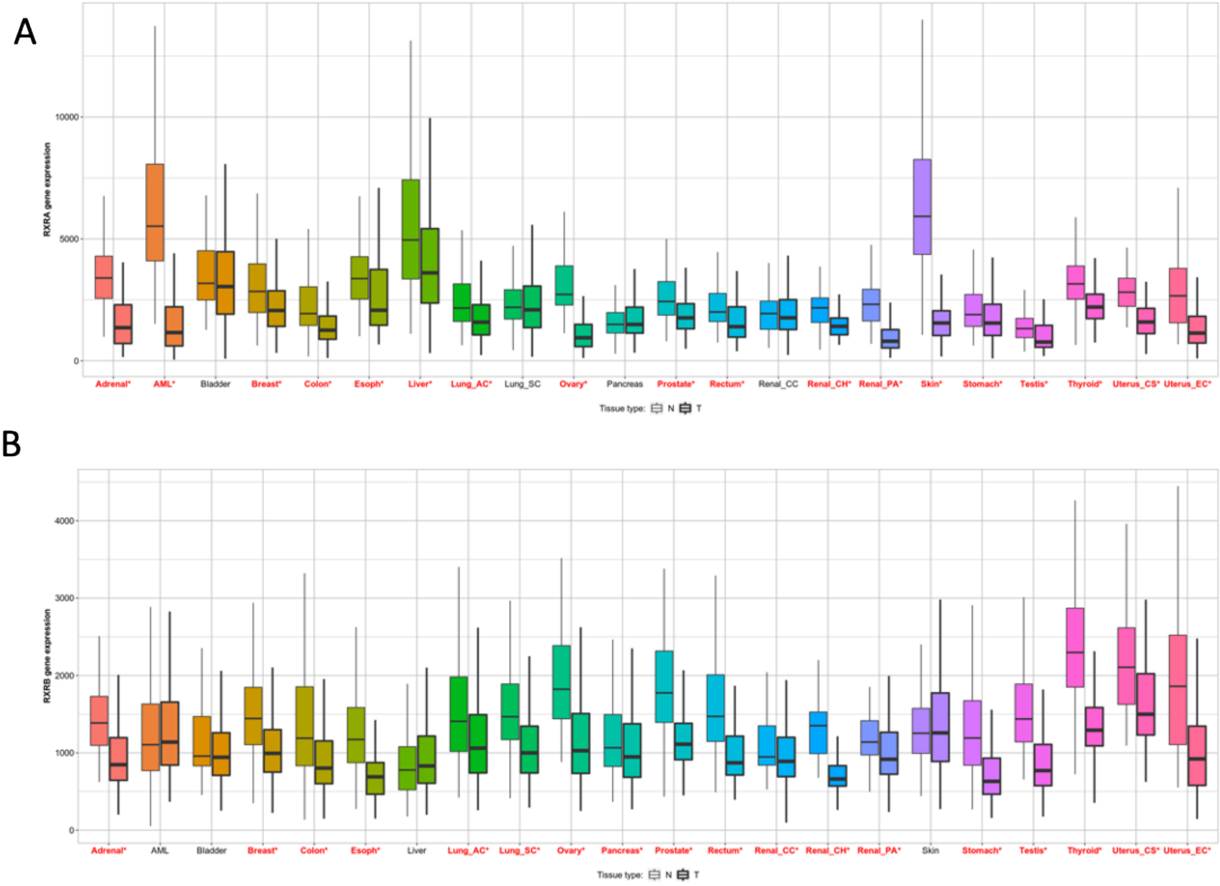


**Figure S1.** Expression of RXR**α** (A) and RXR**β** (B) in tumors vs. normal tissue. Data analyzed/accessed through KMPLOT.

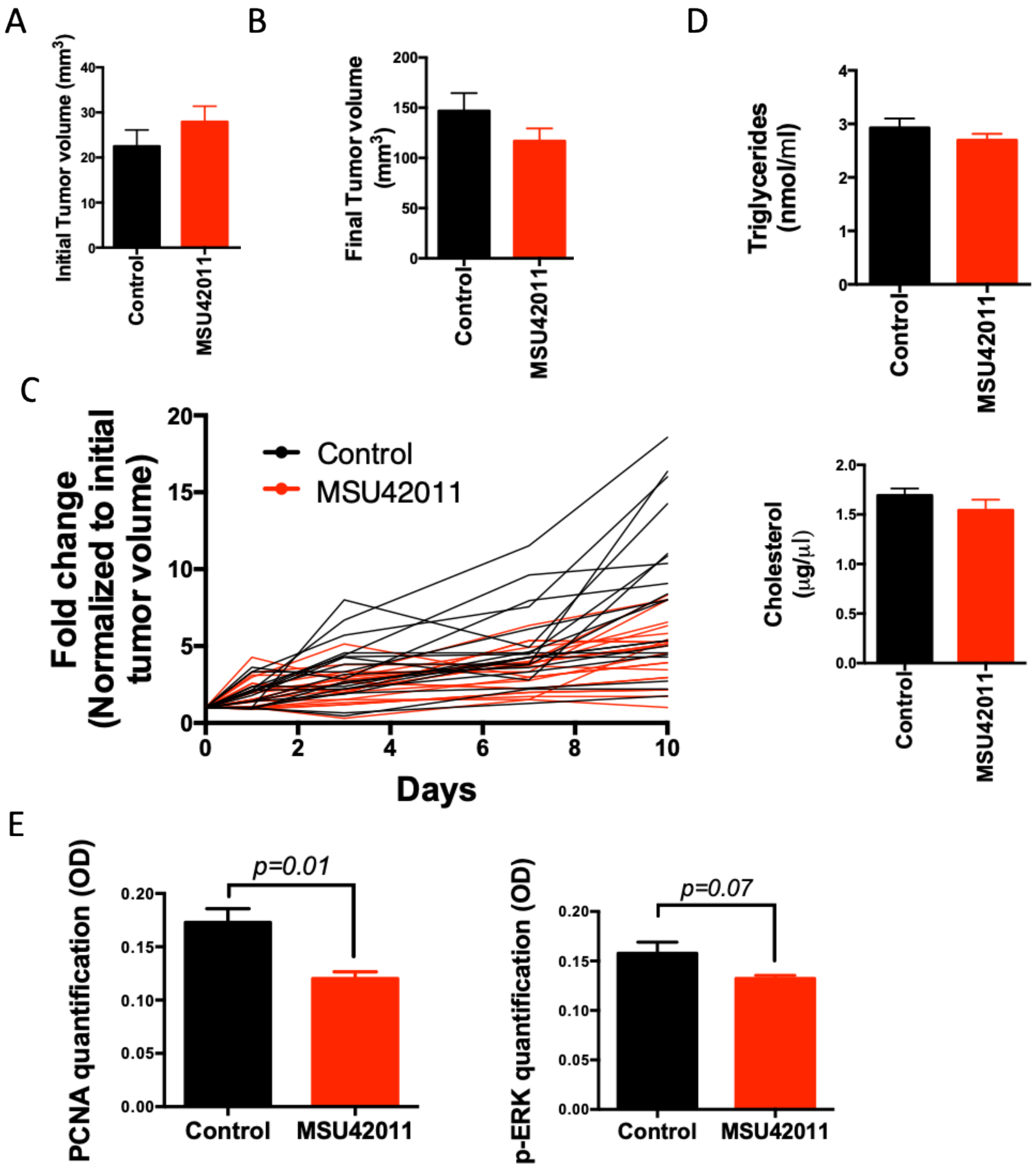


**Figure S2. Tumor volume in MMTV-Neu mice treated with MSU42011.** (A) Initial and (B) final tumor volumes. (C) individual fold changes of tumors over 10 days of treatment. (D) Plasma triglycerides and cholesterol levels in MMTV-Neu after 10-day treatment with MSU42011. (E) Quantification of PCNA and p-ERK intensity staining by optical density (OD), $n$ = 4/group.

.

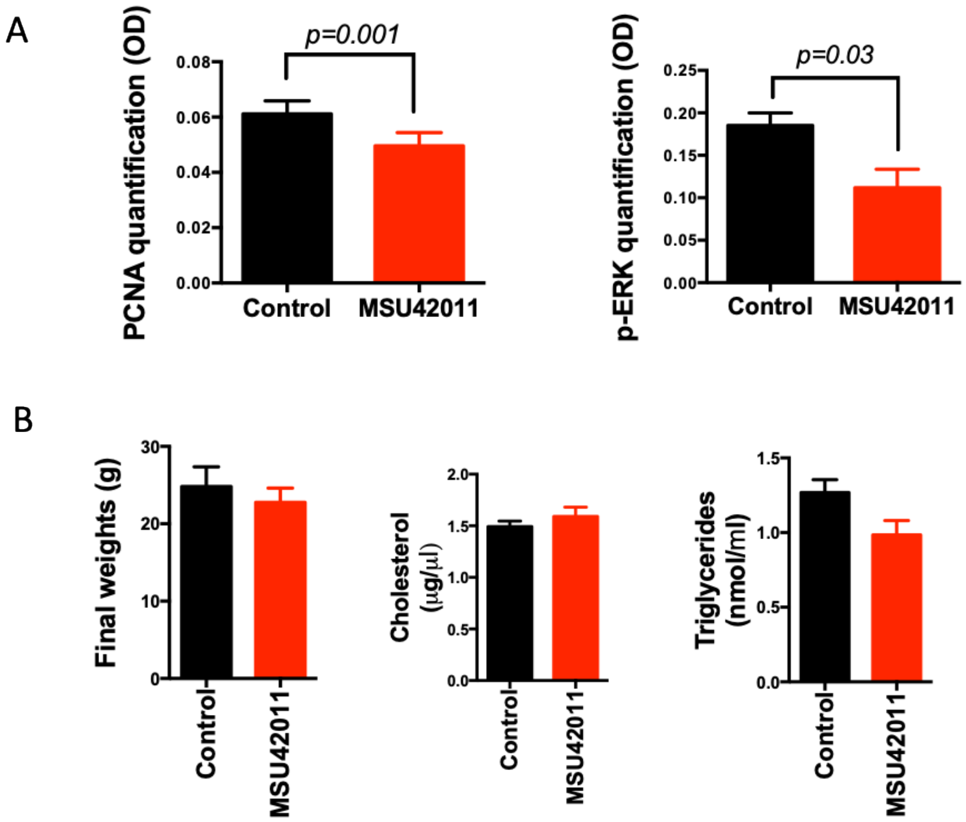


**Figure S3.** (A) Quantification of PCNA (*n* = 3) and p-ERK (*n* = 4) intensity staining by optical density (OD). (B) Final total body weight, plasma triglycerides and cholesterol levels in A/J mice after 12 weeks of treatment with control or MSU42011 (100 mg/Kg).

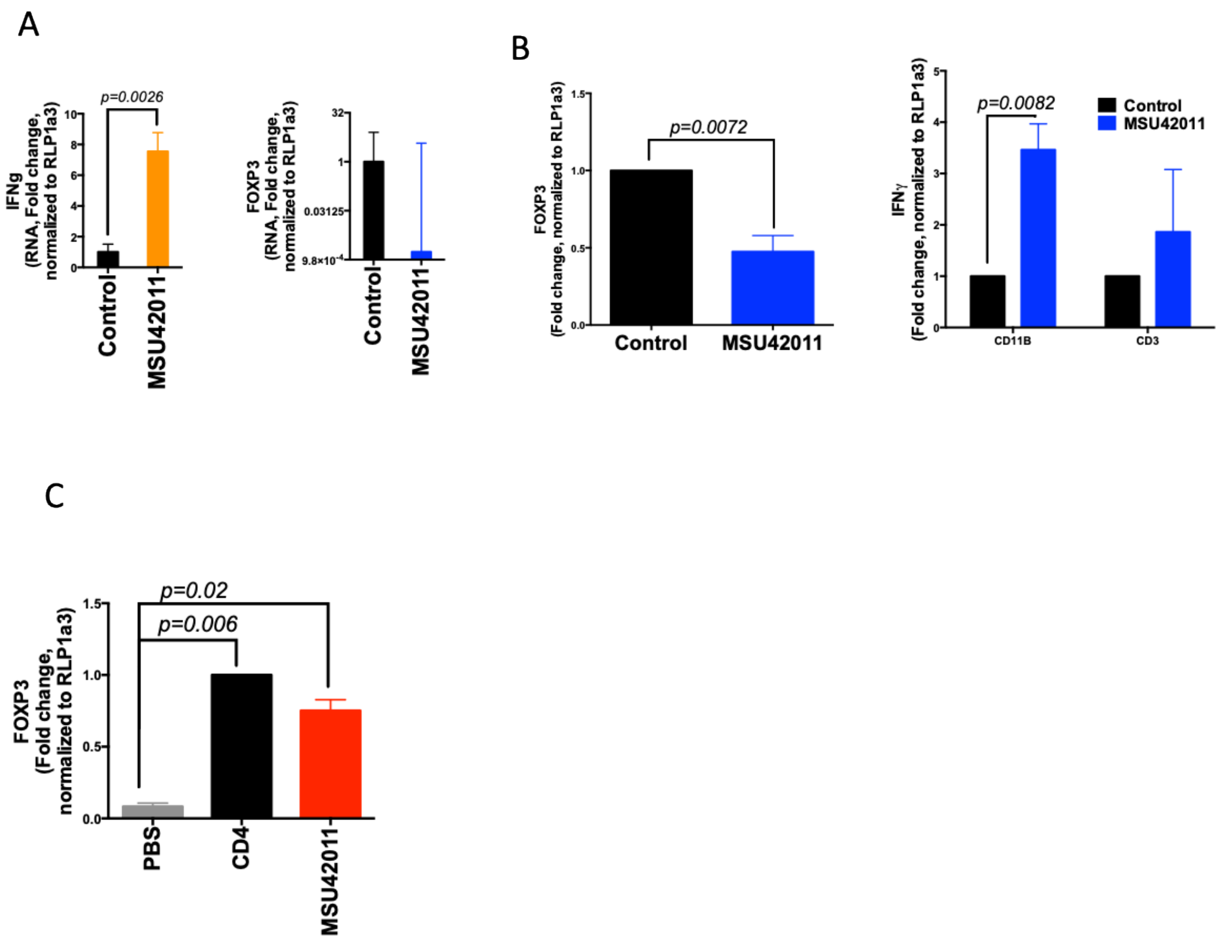


**Figure S4: Treatment with MSU42011 reduced FOXP3 and increased IFNγ.** (A) RT-PCR analysis of whole tumor lysates from MMTV-Neu mice treated with control or MSU42011 diet for 10 days. FOXP3 and IFNγ were evaluated by qPCR and RLP13a was used as control, *n* = 4/group. (B) Magnetically sorted CD3 and CD11b positive cells were collected from MMTV-Neu tumors treated for 10 days with MSU42011 or control diet (*n* = 3 tumors per group). FOXP3 levels were determined in the CD3 population by RT-PCR, which was also used to determine the levels of IFNγ mRNA in both CD3 and CD11b positive populations. (C) In vitro treatment of CD4 T cells isolated from the spleen of mice treated with MSU42011. CD4 T cells were isolated by magnetic bead separation and treated with anti-CD3, CD28, IL-2 and TGFβ for 24 hours alone, to induce a FOXP3 phenotype; MSU42011 was then added, and treatment maintained for 4 additional days. Cells were collected and RT-PCR was used to determine the levels of FOXP3.

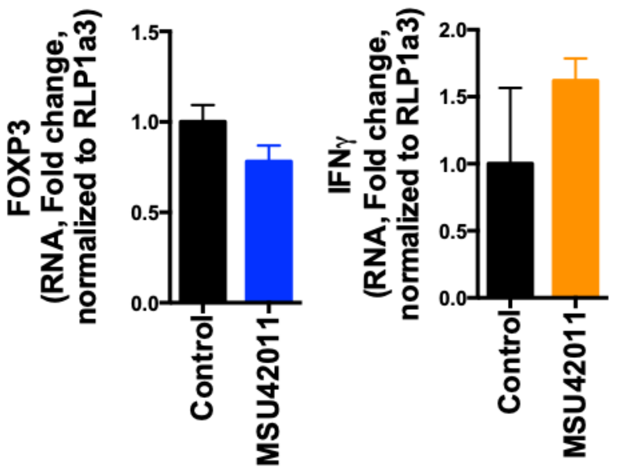


**Figure S5. Evaluation of FOXP3 and increase of IFNγ mRNA in lungs of A/J mice treated with MSU42011.** RT-PCR analysis of whole lung lysates of A/J mice treated with control or MSU42011. FOXP3 and IFNγ were determined by qPCR and RLP13a was used as control, *n* = 5/group.

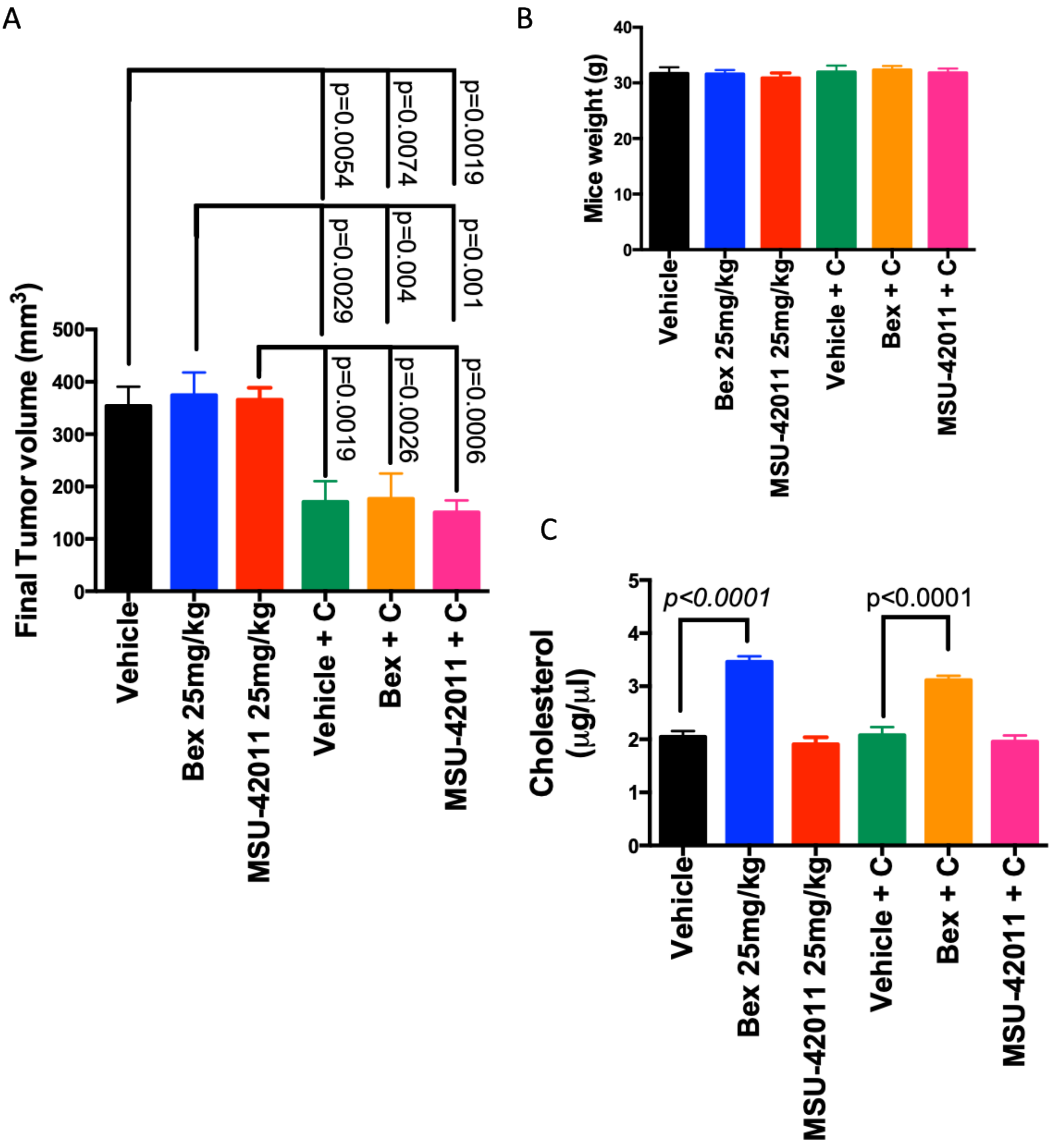


**Figure S6. Nude mice were injected s.c. with human A549 lung cancer cells.** Once a tumor reached 4 mm in diameter, mice were treated as shown and described in Fig 2. (A) Final tumor volumes, (B) average mouse weight, and (C) cholesterol levels in the plasma at the time of necropsy.

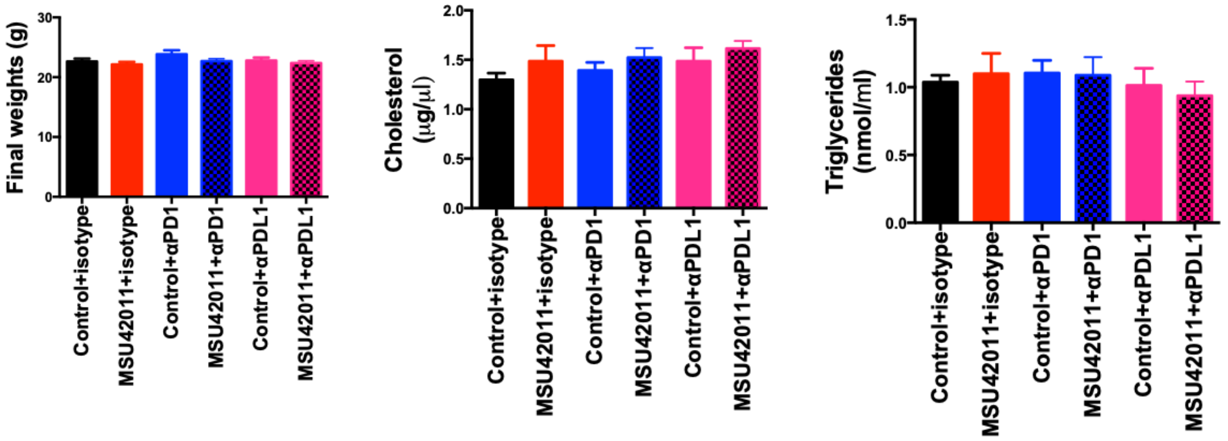


**Figure S7.** Final total body weight, levels of cholesterol and triglycerides in A/J mice treated with control, MSU42011, anti-PD1 or anti-PDL1 antibodies, or the combinations as described in Fig. 7. .

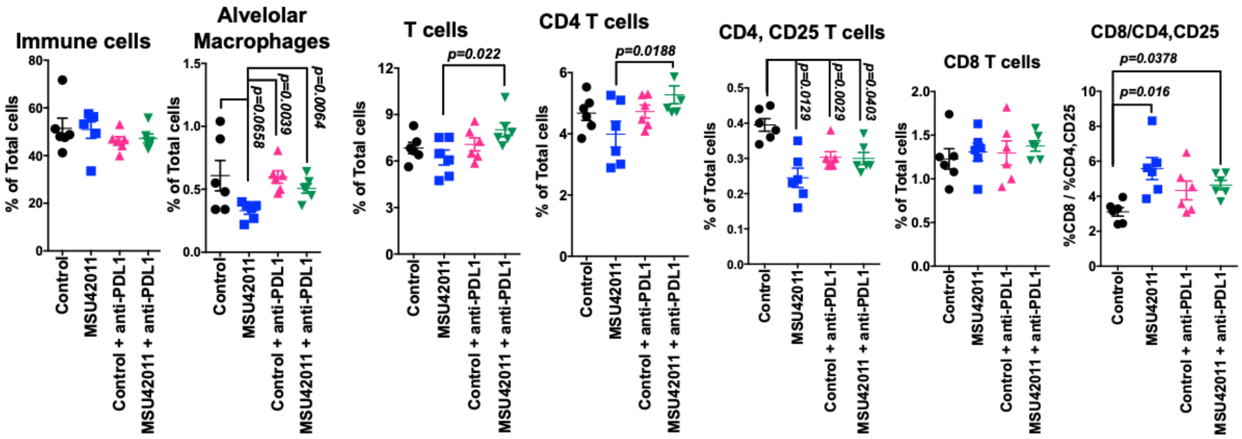


**Figure S8.** Flow cytometry analysis of selected groups of A/J mice treated as described in Fig.7.
